# Supplementary material for: Dichotomous outcomes of TNFR1 and TNFR2 signaling in NK cell-mediated immune responses during inflammation
Source: Nat Commun. 2024 Nov 14;15:9871. doi: 10.1038/s41467-024-54232-y (PMC11564688; doi:10.1038/s41467-024-54232-y)
Supplement: Supplementary file 1 — Supplementary Information [file 41467_2024_54232_MOESM1_ESM.pdf]

Supplementary Information

Dichotomous outcomes of TNFR1 and TNFR2 signaling in NK cell-mediated immune responses during inflammation

Timothy R. McCulloch[#*], Gustavo R. Rossi[#], Louisa Alim[#], Pui Yeng Lam, Joshua Wong, Elaina Coleborn, Snehlata Kumari, Colm Keane, Andrew J. Kueh, Marco J. Herold, Christoph Wilhelm, Percy A. Knolle, Lawrence Kane, Timothy J. Wells, and Fernando Souza-Fonseca-Guimaraes[*]

[#] These authors contributed equally

[*] Correspondence: Fernando Souza-Fonseca-Guimaraes and Timothy R. McCulloch

E-mails: f.guimaraes@uq.edu.au and timothym@uni-bonn.de

Address: 37 Kent St, Woolloongabba, QLD 4102, Australia

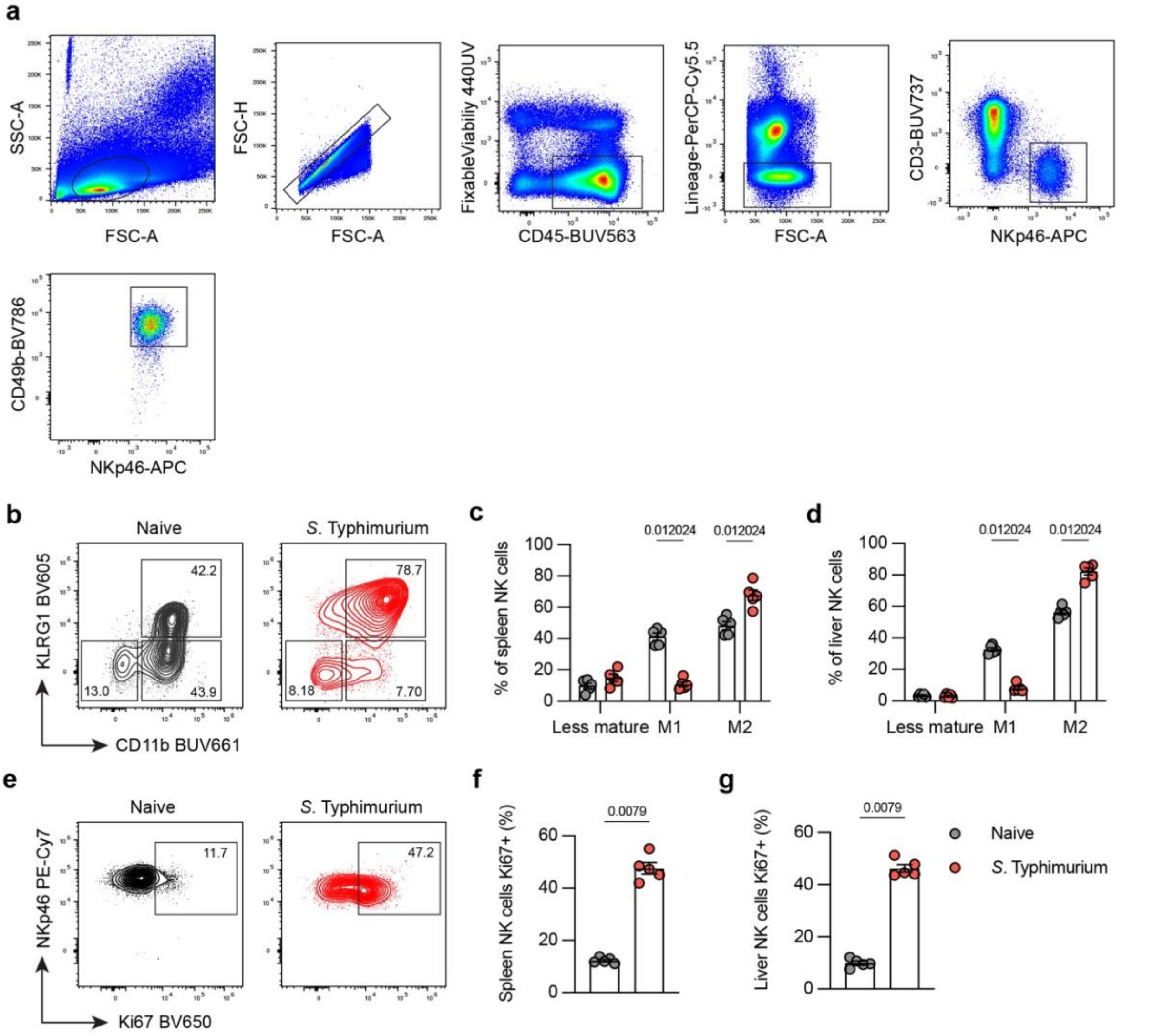

Supplementary Fig. 1: Characterizing NK cells during *S*. Typhimurium infection. Wild-type C57BL/6 mice were infected with attenuated *S*. Typhimurium (SL3261) and NK cells analyzed in the spleens and livers at day four post infection. **a**, Representative gating strategy used throughout the paper to examine NK cells by flow cytometry. **b**, Representative flow cytometry plots showing spleen NK cell maturation, where "less mature" are defined as CD11b$^-$KLRG1$^-$, M1 defined as CD11b$^+$KLRG1$^-$, and M2 defined as CD11b$^+$KLRG1$^+$. **c,d**, Relative proportions of each NK cell maturation state in the spleen (**c**) or liver (**d**). **e**, Representative flow cytometry plots showing proliferation by Ki67 expression in spleen NK cells. **f,g**, Relative proportions of NK cells expressing Ki67 the spleen (**c**) or liver (**d**). Data in **b-g** from a single experiment (*n* = 5 biological replicates). Error bars indicate mean ± SEM. Groups were compared using two-tailed Mann-Whitney U test, where $P < 0.05$ was considered statistically significant. Source data are provided as a Source Data file.

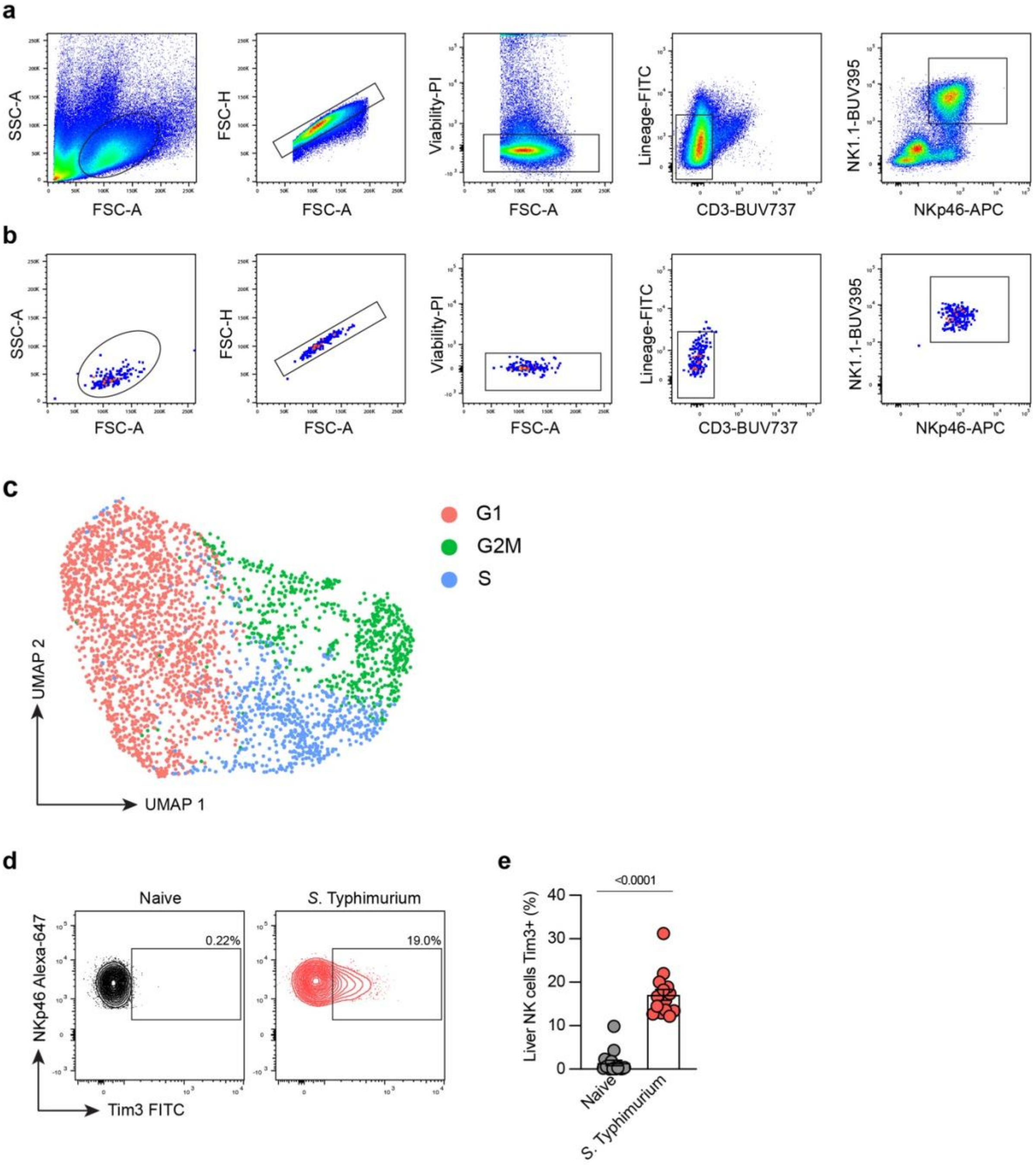

Supplementary Fig. 2: Tim3 is upregulated on NK cells during *S*. Typhimurium infection. Wild-type C57BL/6 mice were infected with attenuated *S*. Typhimurium (SL3261) and NK cells pre-enriched and sorted from the spleens at day four post infection. **a,** Gating strategy to sort pre-enriched NK cells. **b,** Representative post-sort purity of NK cells for scRNA-seq. **c**, Cell Cycle Scoring of NK cells from scRNA-seq. Wild-type C57BL/6 mice were infected with attenuated *S*. Typhimurium (SL3261) and NK analyzed at day four post infection. **d**, Representative flow plots showing expression of Tim3 on liver NK cells. **e**, Percentage of NK cells expressing Tim3 in the liver of naïve or *S*. Typhimurium infected mice. Data in **a-c** from a single experiment (*n* = 4 biological replicates) and **d,e** pooled from two independent experiments (*n* = 15 naïve mice and 14 *S*. Typhimurium infected). Error bars indicate mean ± SEM. Groups were compared using two-tailed Mann-Whitney U test, where $P < 0.05$ was considered statistically significant. Source data are provided as a Source Data file.

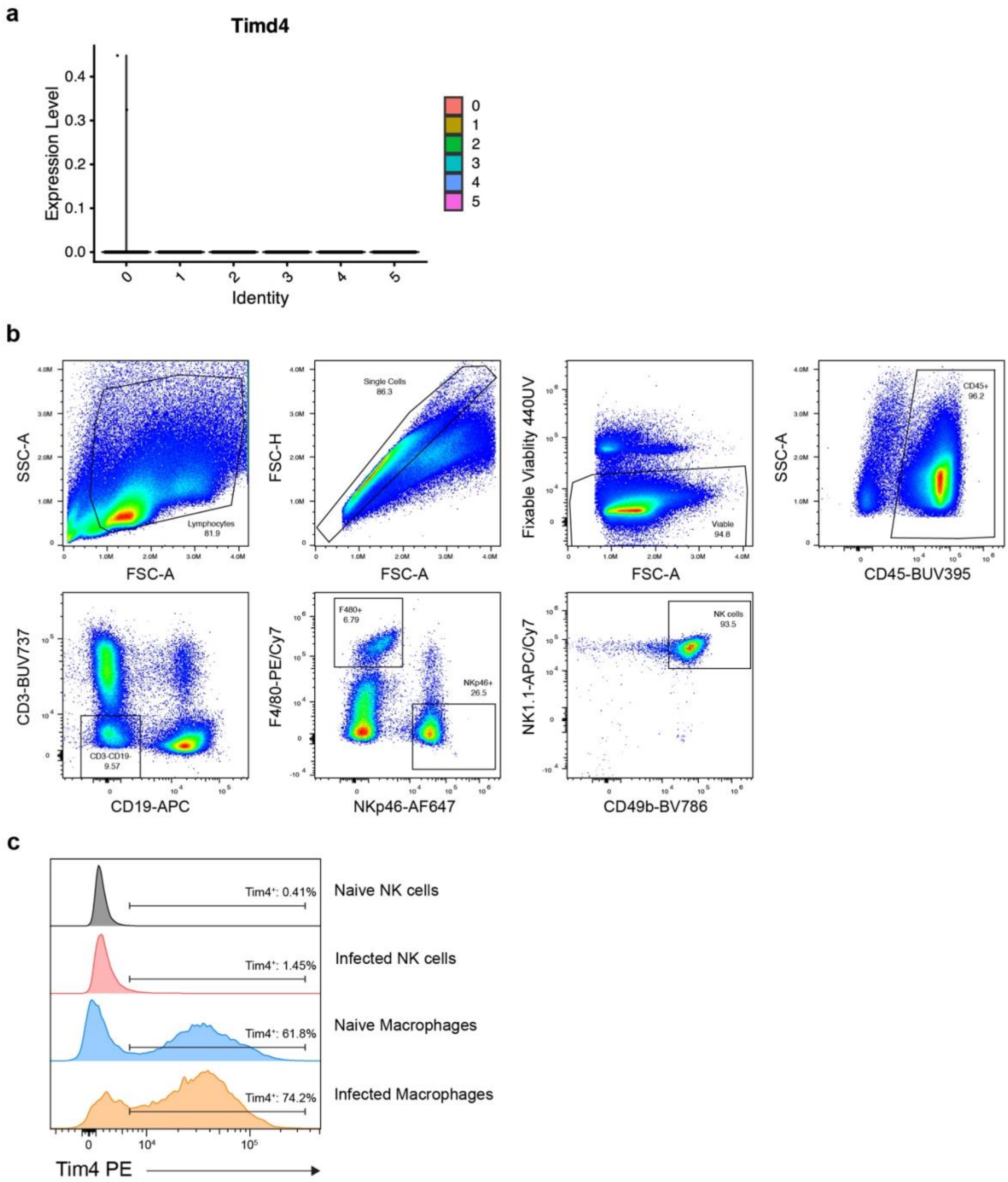

Supplementary Figure 3: Tim4 is not expressed in NK cell populations. Wild-type C57BL/6 mice were infected with *S*. Typhimurium and NK cells analyzed by scRNA-seq on day four post-infection. **a**, Expression of *Timd4* (encoding Tim4) across NK cell clusters. NK cells and F4/80^{+} cells (assumed to be macrophages) from C57BL/6 spleens were analyzed by flow cytometry at day four post infection. **b**, Representative gating strategy to examine NK cells and F4/80^{+} macrophages. **c**, Expression of Tim4 in indicated cell types. Data from a single experiment, representative of 5 biological replicates.

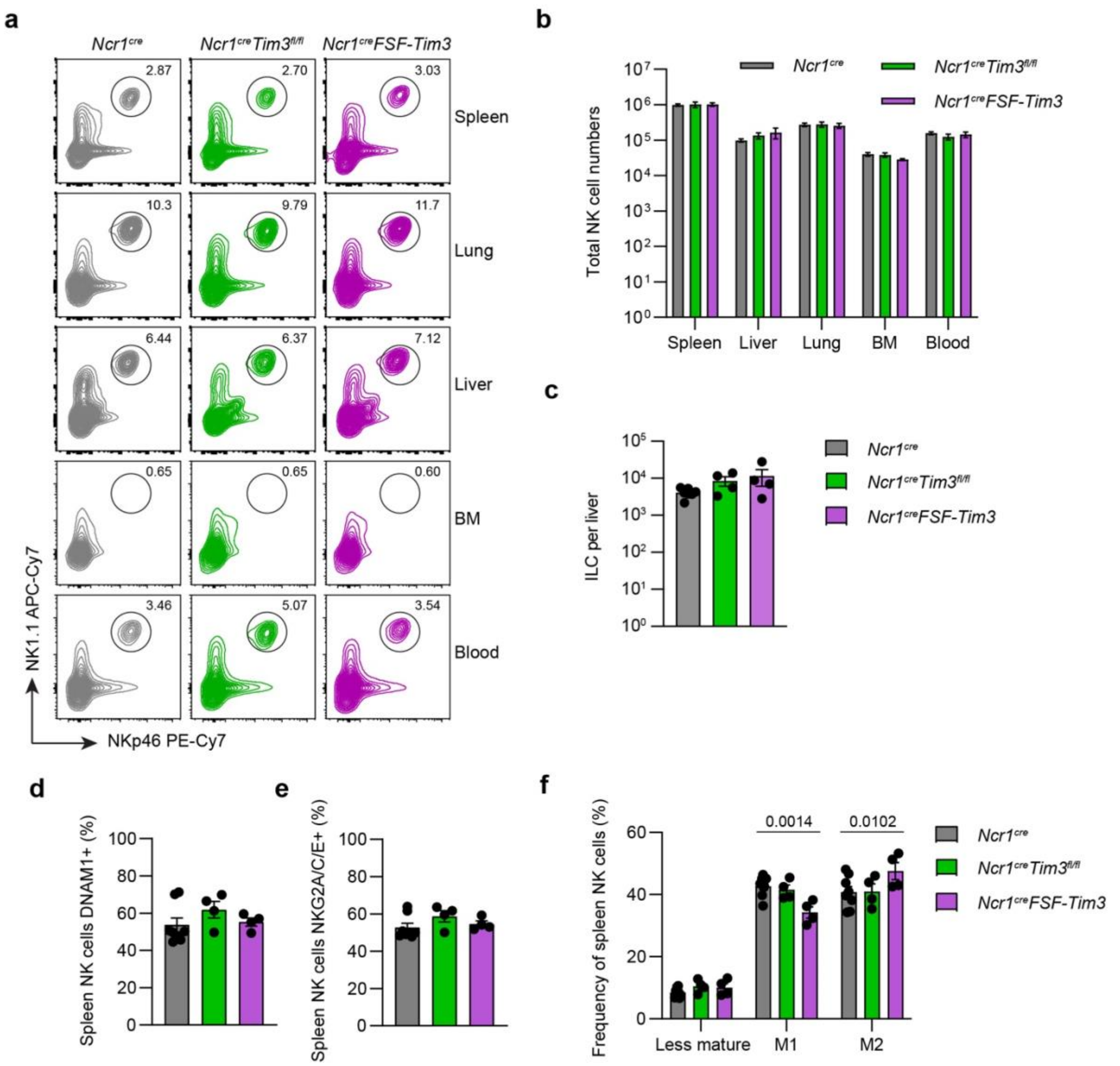

Supplementary Fig. 4: Development of NK cells is largely normal in transgenic Tim3 mice. Various organs were taken from naïve *Ncr1^{cre}* (wild-type), *Ncr1^{cre}Tim3$^{fl/fl}$* (Tim3-null), or *Ncr1^{cre}FSF-Tim3* (Tim3-overexpression) to analyze NK cell populations. **a**, Representative flow cytometry plots showing frequency of NK cells in the indicated organ. **b**, Quantification of total NK cell numbers per organ (blood represented as NK cells/ml). **c**, Quantification of total ILC1 numbers in the liver. **d-e**, Expression of NK maturation markers DNAM1 and NKG2A/C/E. **f**, Maturation of spleen NK cells determined by CD11b and KLRG1 staining (Less mature = CD11b$^-$KLRG1$^-$, M1 = CD11b$^+$KLRG1$^-$, M2 = CD11b$^+$KLRG1$^+$). Data are from a single experiment (n = 8 *Ncr1^{cre}*, 4 *Ncr1^{cre}Tim3$^{fl/fl}$*, and 4 *Ncr1^{cre}FSF-Tim3*). Each dot represents one animal, error bars indicate mean ± SEM. Groups were compared using 2-way ANOVA with Dunnett's multiple comparisons test for **b** and **f**, or Kruskal-Wallis test with Dunn's multiple comparisons test for **c-e**, where $P < 0.05$ was considered statistically significant. Abbreviations: BM, bone marrow; ILC, innate lymphoid cell. Source data are provided as a Source Data file.

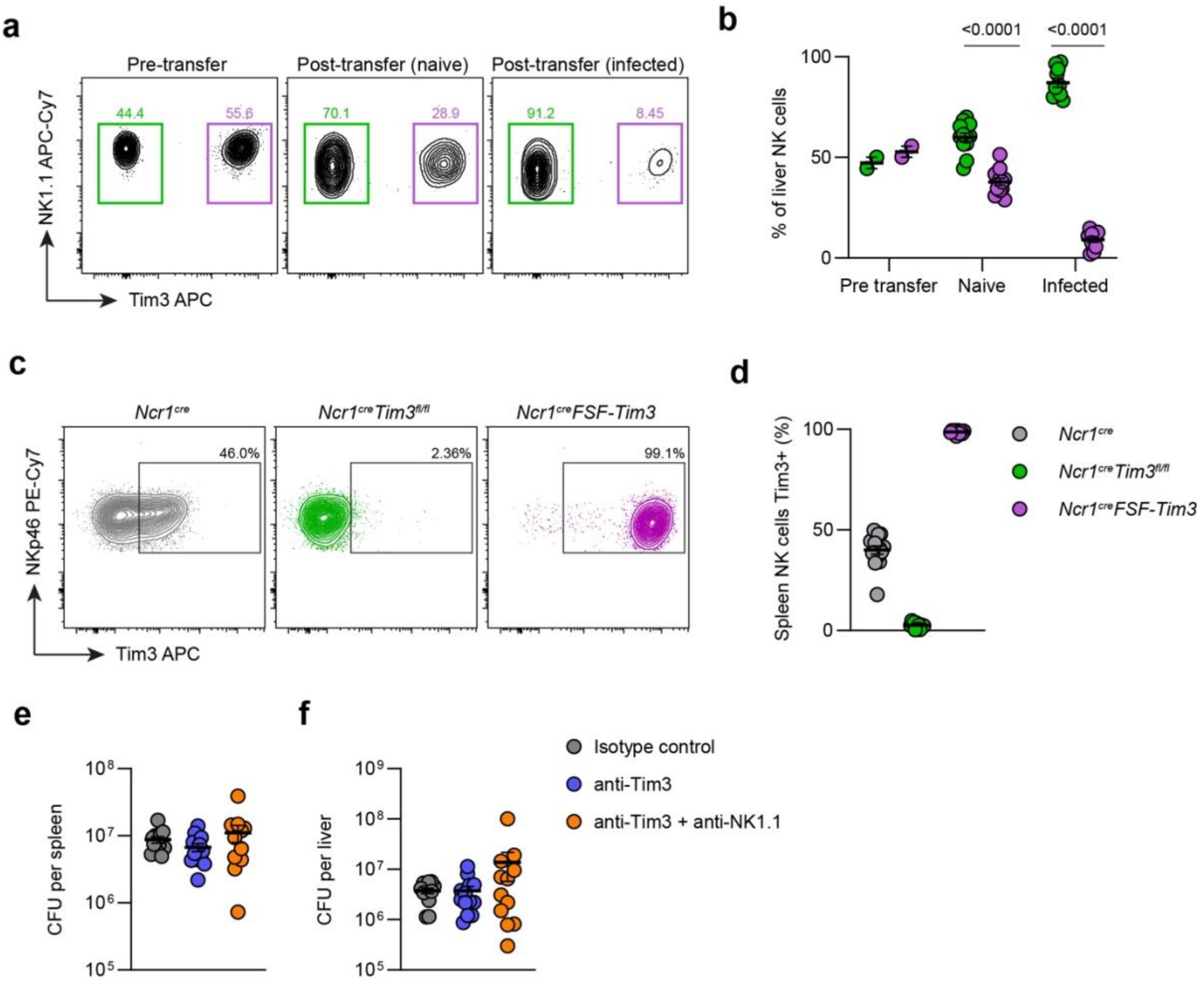

Supplementary Fig. 5: Overexpression of Tim3 restricts NK cell accumulation and function. NK cells isolated from Tim3-null or Tim3-overexpressing mice were adoptively transferred into *Rag2*$^{-/-}$*γc*$^{-/-}$ mice and recipient mice infected or left naïve. **a**, Representative flow cytometry plots showing proportions of Tim3-null and Tim3-overexpressing NK cells pre-transfer and in adoptively transferred mice with or without infection. **b**, Relative proportions of NK cells pre-transfer and in livers of adoptively transferred mice with or without infection. *Ncr1*^{cre}, *Ncr1*^{cre}*Tim3*$^{fl/fl}$, and *Ncr1*^{cre}*FSF-Tim3* mice were infected with *S.* Typhimurium and spleens and livers analyzed at day four post infection. **c**, Representative flow cytometry plots showing expression of Tim3 on splenic NK cells. **d**, Expression of Tim3 on splenic NK cells. C57BL/6 mice were infected with *S*. Typhimurium and treated with anti-Tim3 blocking antibodies with or without NK cell depletion. **e,f**, Bacterial burdens in the spleen (**e**) and liver (**f**) of treated mice. Data are from two independent experiments (**a,b**: *n* = 12 naïve mice and 9 *S.* Typhimurium infected; **c-d**: *n* = 15 *Ncr1*^{cre}, 12 *Ncr1*^{cre}*Tim3*$^{fl/fl}$, and 9 *Ncr1*^{cre}*FSF-Tim3*; **e,f**: *n* = 12 isotype control treated mice, 13 anti-Tim3 treated mice, and 12 anti-Tim3 + anti-NK1.1 treated mice). Each dot represents one animal, error bars indicate mean ± SEM. Groups were compared using two-tailed Mann-Whitney U test for **b**, or Kruskal-Wallis test with Dunn's multiple comparisons test for **e,f**, where $P < 0.05$ was considered statistically significant. Abbreviations: CFU, colony forming units. Source data are provided as a Source Data file.

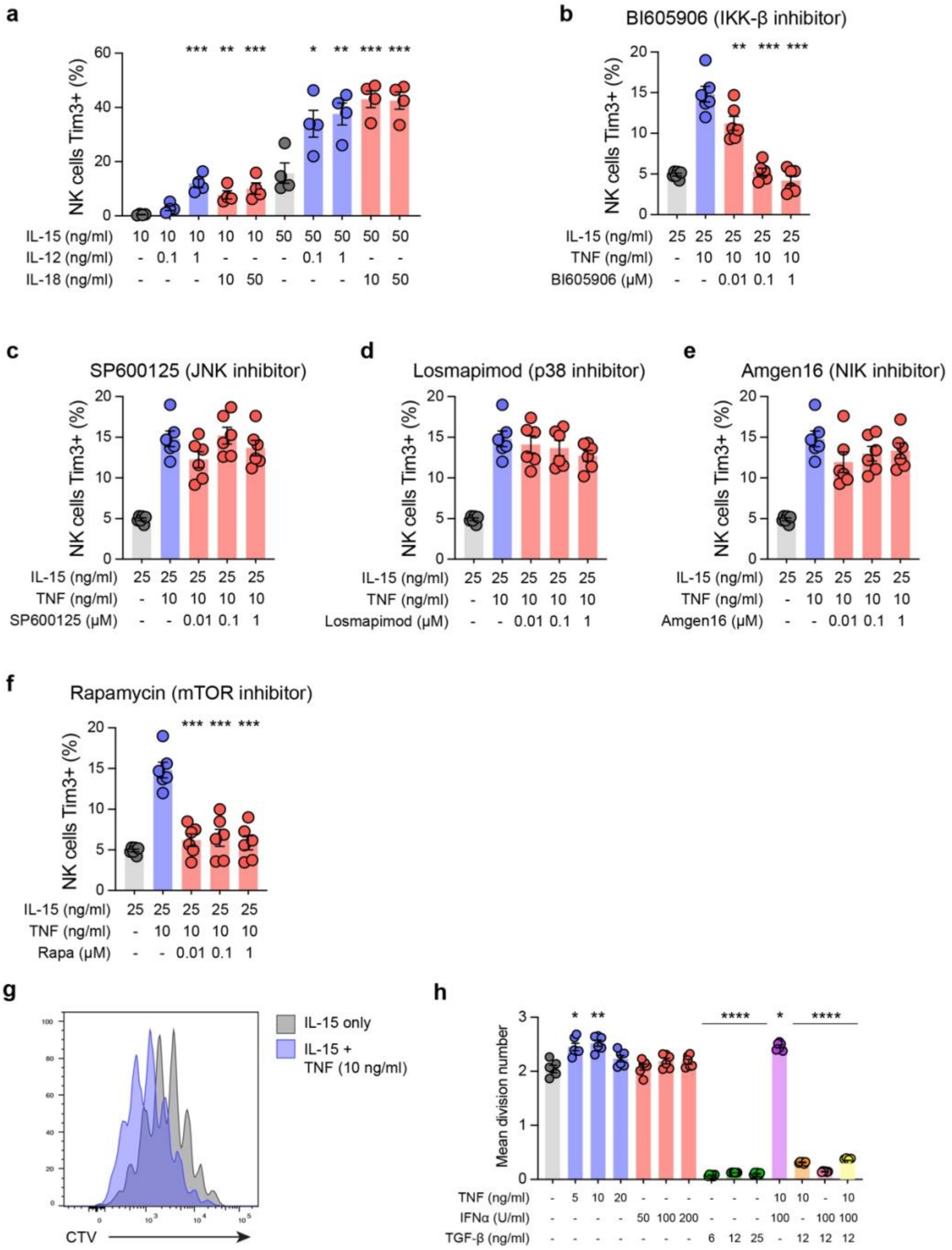

Supplementary Fig. 6: Upregulation of Tim3 in NK cells. NK cells from wild-type C57BL/6 mice were isolated and cultured for three days in the presence of the indicated cytokines and/or inhibitors. **a**, Expression of Tim3 on NK cells in response to IL-12 or IL-18 signaling. **b**, Effects of IKK-β inhibitor BI605906 on TNF-induced Tim3 expression. **c**, Effects of JNK inhibitor SP600125 on TNF-induced Tim3 expression. **d**, Effects of p38 inhibitor Losmapimod on TNF-induced Tim3 expression. **e**, Effects of NIK inhibitor Amgen16 on TNF-induced Tim3 expression. **f**, Effects of mTOR inhibitor rapamycin on TNF-induced Tim3 expression. **g**, Representative flow cytometry plots showing CTV dye dilution. **h**, Mean division number, calculated as described by Hawkins *et al*[28]. Data are from two independent experiments (n = 6 biological replicates). Each dot represents one animal, error bars indicate mean ± SEM. In **a** and **h** groups were compared to IL-15 only, and in **b-f** groups were compared to IL-15 + TNF using one-way ANOVA with Dunnett's multiple comparisons test (*$P < 0.05$, **$P < 0.01$, ***$P < 0.001$), where $P < 0.05$ was considered statistically significant. Abbreviations: TNF, tumor necrosis factor; IFN, interferon; TGF, tumor growth factor. Source data are provided as a Source Data file.

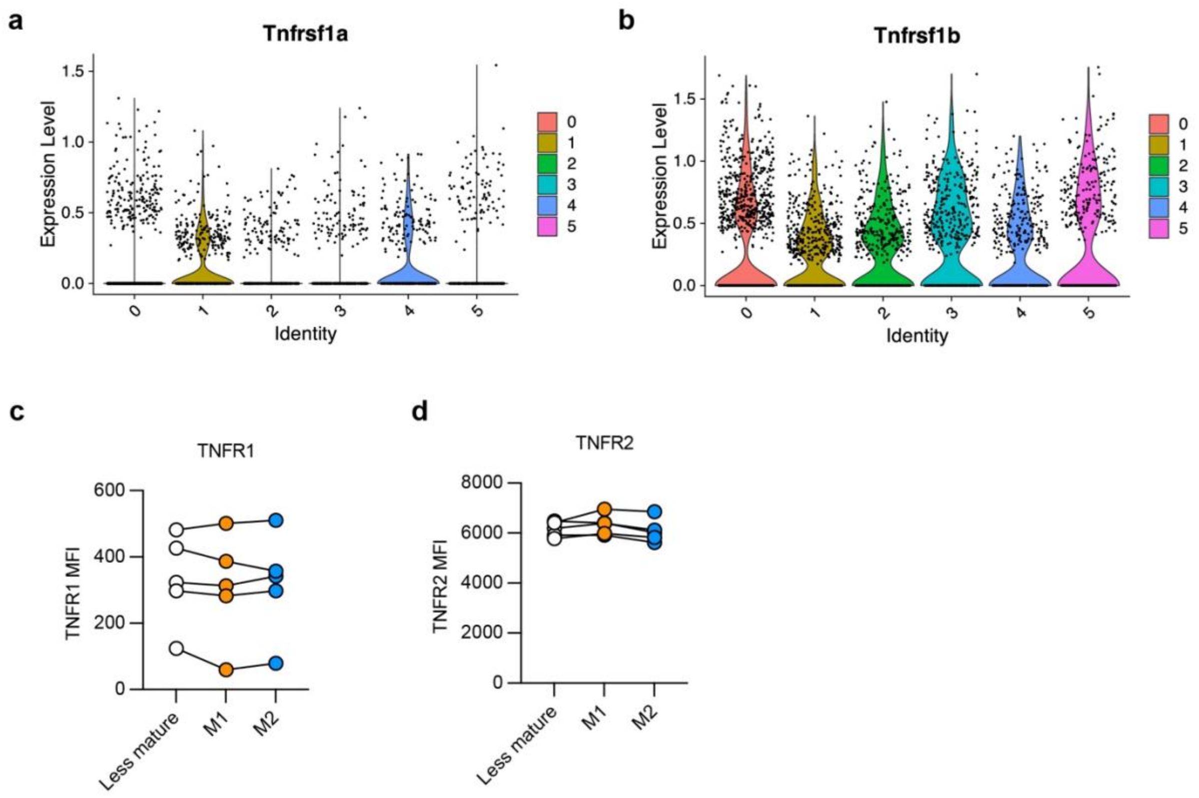

Supplementary Fig. 7: TNFR expression across NK cell populations. Wild-type mice were infected with *S*. Typhimurium and NK cells analyzed by scRNA-seq on day four post-infection. **a**, Expression of *Tnfrsf1a*, encoding TNFR1, across NK cell clusters. **b**, Expression of *Tnfrsf1b*, encoding TNFR2, across NK cell clusters. Splenic NK cells from naïve mice were analyzed by flow cytometry. c, MFI of TNFR1 across NK cell populations. d, MFI of TNFR2 across NK cell populations (less mature: $CD11b^-KLRG1^-$; M1: $CD11b^+KLRG1^-$; $CD11b^+KLRG1^+$). Source data are provided as a Source Data file. Data from a single experiment (*n* = 5 biological replicates). Source data are provided as a Source Data file.

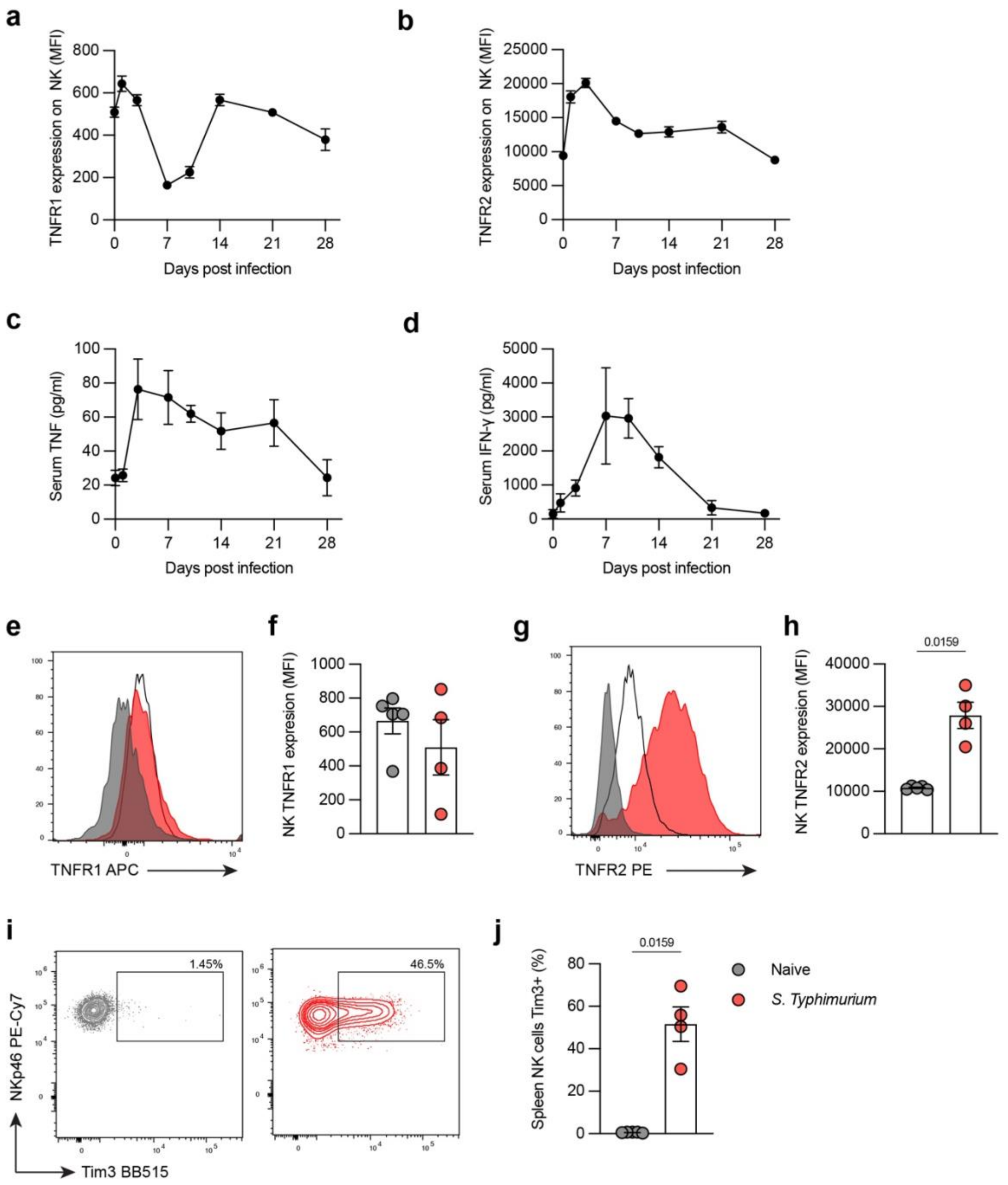

Supplementary Fig. 8: Expression of TNFR1 and TNFR2 on NK cells during *S.* Typhimurium infection. Wild-type C57BL/6 mice were infected with attenuated *S*. Typhimurium (SL3261) and splenic NK cells analyzed by flow cytometry at different timepoints post-infection. **a**, Expression of TNFR1 on NK cells at different timepoints. **b**, Expression of TNFR2 on NK cells at different timepoints. **c**, Serum titers of IFN-γ at different timepoints. **d**, Serum titers of TNF at different timepoints. Wild-type C57BL/6 mice were infected with wild-type *S*. Typhimurium (SL1344) by oral gavage and NK cells from all animals analyzed three days post onset of weight loss was observed in any mouse. **e-f,** Expression of TNFR1 on splenic NK cells. **g-h,** Expression of TNFR2 on splenic NK cells. **i-j**, Expression of Tim3 on splenic NK cells. Data from a single experiment (**a,b:** n = 4-5 biological replicates per timepoint; **c,d:** 3-7 biological replicates per timepoint; **e-j**: n = 5 naïve mice and 4 *S.* Typhimurium infected) Error bars indicate mean ± SEM. Groups were compared using two-tailed Mann-Whitney U test, where $P < 0.05$ was considered statistically significant. Source data are provided as a Source Data file.

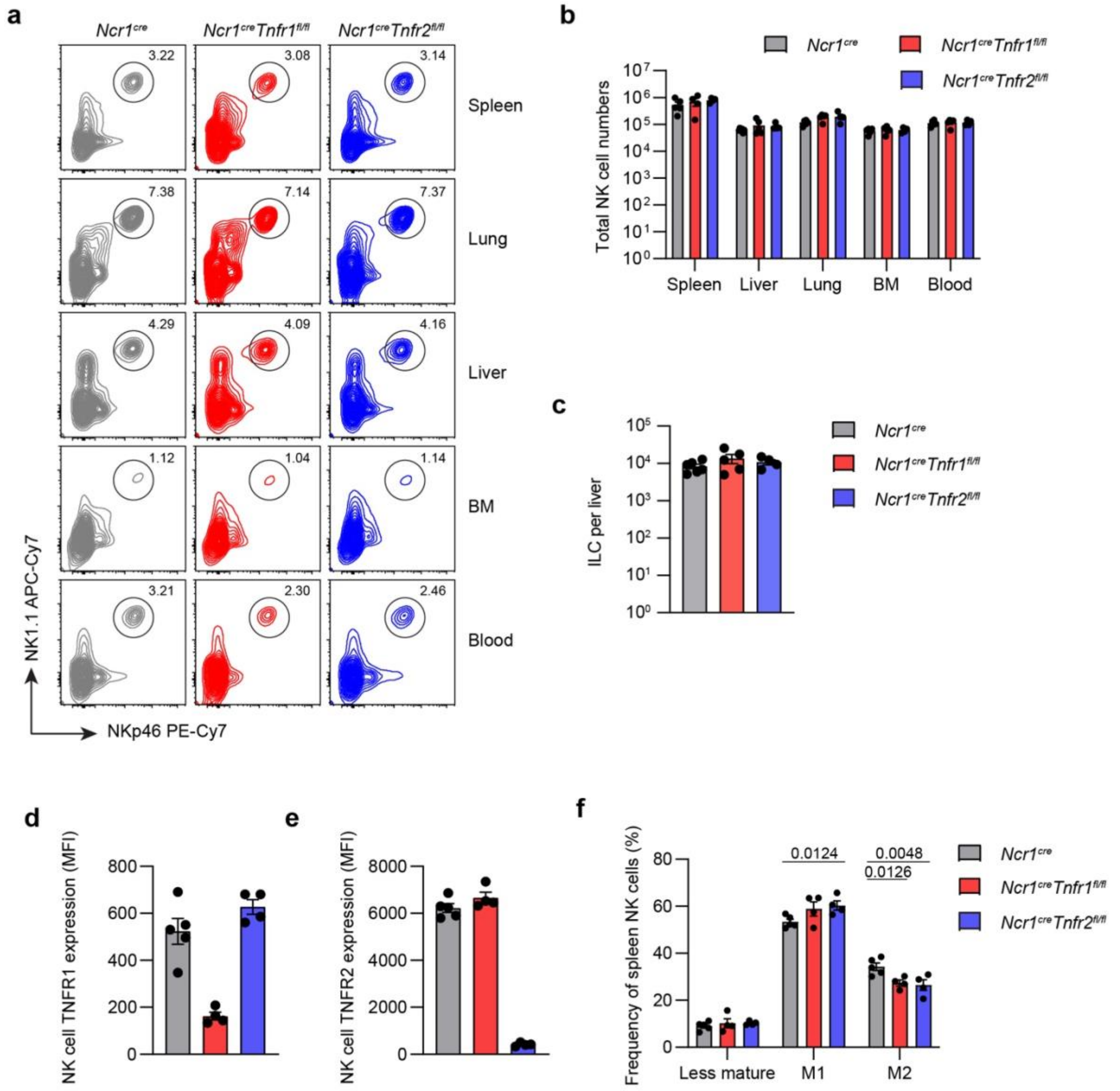

Supplementary Fig. 9: Normal NK cell development during conditional deletion of TNFR1 or 2 in NK cells. Various organs were taken from naïve $Ncr1^{cre}$, $Ncr1^{cre}Tnfr1^{fl/fl}$, or $Ncr1^{cre}Tnfr2^{fl/fl}$ to analyze NK cell populations. **a**, Representative flow cytometry plots showing frequency of NK cells in the indicated organ. **b**, Quantification of total NK cell numbers per organ (blood represented as NK cells/ml). **c**, **b**, Quantification of total ILC1 numbers per liver. **d,e**, Expression of TNFR1 (**d**) or TNFR2 (**e**) in splenic NK cells **f**, Maturation of spleen and NK cells determined by CD11b and KLRG1 staining (Less mature = $CD11b^{-}KLRG1^{-}$, M1 = $CD11b^{+}KLRG1^{-}$, M2 = $CD11b^{+}KLRG1^{+}$). Data are from a single experiment (n = 5 $Ncr1^{cre}$, 4 $Ncr1^{cre}Tnfr1^{fl/fl}$, and 4 $Ncr1^{cre}Tnfr2^{fl/fl}$). Each dot represents one animal, error bars indicate mean ± SEM. Groups were compared using Kruskal-Wallis test with Dunn's multiple comparisons test, where $P < 0.05$ was considered statistically significant. Abbreviations: BM, bone marrow; ILC, innate lymphoid cell. Source data are provided as a Source Data file.

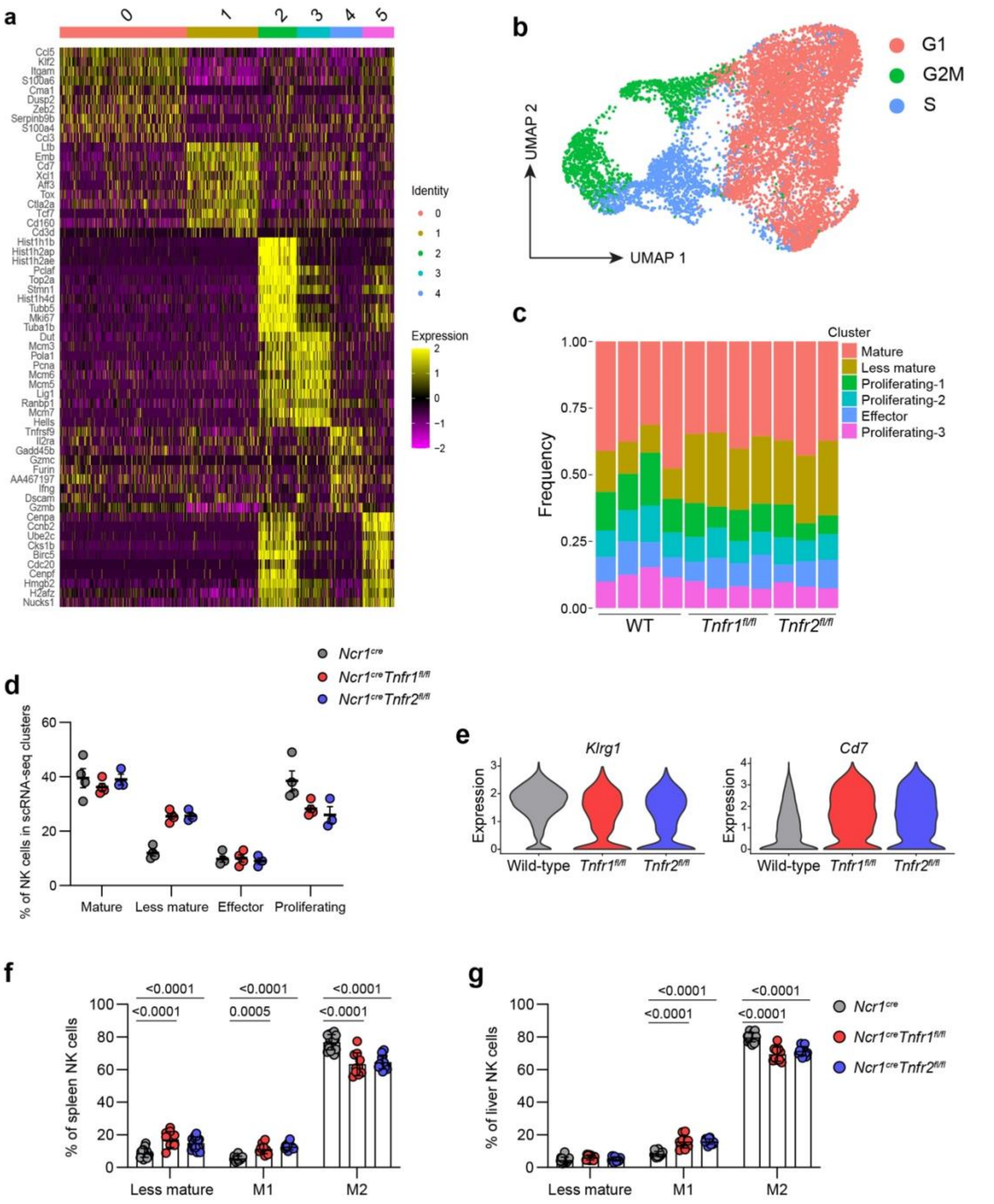

Supplementary Fig. 10: scRNA-seq of NK cells from transgenic TNF receptor mice. *Ncr1*^{cre}, *Ncr1*^{cre}*Tnfr1*$^{fl/fl}$, or *Ncr1*^{cre}*Tnfr2*$^{fl/fl}$ were infected with *S*. Typhimurium and NK cells pre-enriched and sorted at day four post-infection for scRNA-seq. **a**, Heatmap showing top 10 signature genes identifying each Seurat cluster. **b**, Cell cycle prediction. **c**, Relative frequency of NK cells in each Seurat cluster, where each column represents a biological replicate. **d**, Percentage of NK cells in each Seurat cluster (proliferating 1, 2, and 3 combined). **e**, Violin plots showing relative expression of *Klrg1* or *Cd7*, **f**, Percentage of splenic NK cells in each maturation stage based on CD11b and KLRG1 expression (Imm = CD11b$^-$KLRG1$^-$, M1 = CD11b$^+$KLRG1$^-$, M2 = CD11b$^+$KLRG1$^+$). Data from **a-e** from a single experiment (n = 4 *Ncr1*^{cre}, 4 *Ncr1*^{cre}*Tnfr1*$^{fl/fl}$, and 3 *Ncr1*^{cre}*Tnfr2*$^{fl/fl}$), and data from **f-g** pooled from two independent experiments (n = 17 *Ncr1*^{cre}, 10 *Ncr1*^{cre}*Tnfr1*$^{fl/fl}$, and 15 *Ncr1*^{cre}*Tnfr2*$^{fl/fl}$). Each dot represents one animal, error bars indicate mean ± SEM. Groups were compared using Kruskal-Wallis test with Dunn's multiple comparisons test, where $P < 0.05$ was considered statistically significant. Source data are provided as a Source Data file.

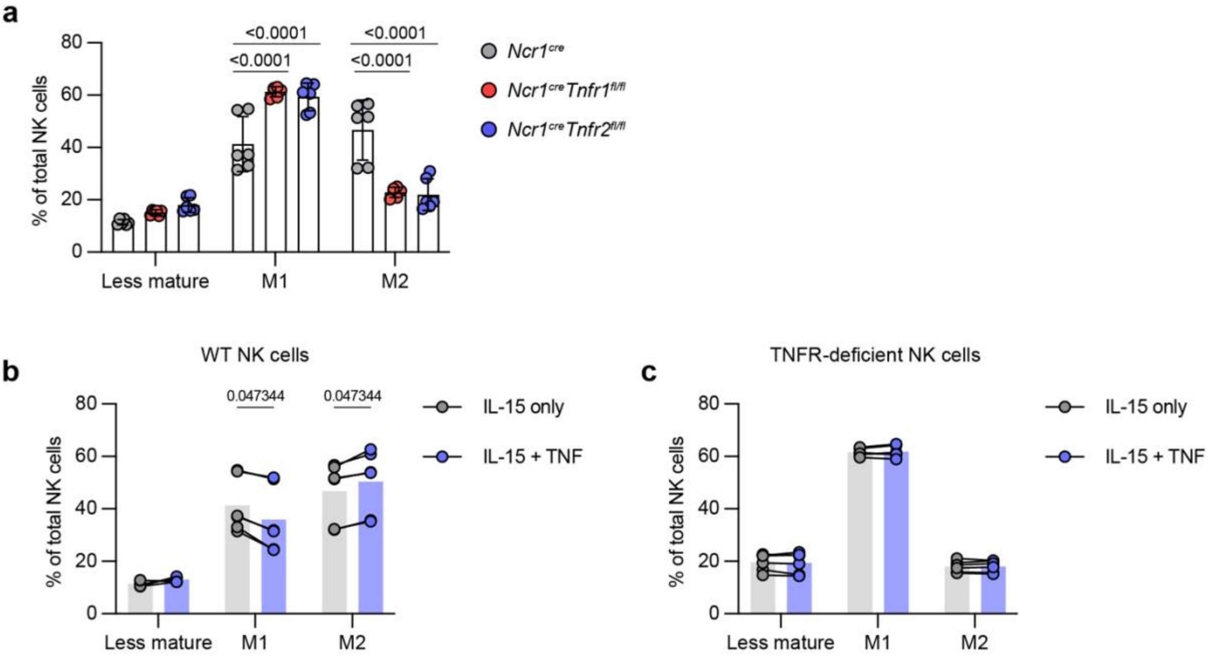

Supplementary Fig. 11: Maturation of TNFR-deficient NK cells. NK cells isolated from indicated genotypes were cultured in the presence of 25 ng/ml IL-15 +/- 10 ng/ml TNF for 3 days, then CD11b and KLRG1 expression measured to determine maturation status (less mature: CD11b$^-$KLRG1$^-$; M1: CD11b$^+$KLRG1$^-$; M2: CD11b$^+$KLRG1$^+$). **a**, Maturation status of each genotype treated with 25 ng/ml IL-15 only. **b**, Maturation status of NK cells from WT mice in response to IL-15 +/- TNF. **c**, Maturation status of NK cells from TNFR1/TNFR2-floxed mice in response to IL-15 +/- TNF. Data are from two independent experiments (*n* = 6 biological replicates). Each dot represents one animal, error bars indicate mean ± SEM. Groups were compared using Kruskal-Wallis test with Dunn's multiple comparisons test for **a**, or Wilcoxon matched-pairs rank test for **b-c**, where $P < 0.05$ was considered statistically significant. Source data are provided as a Source Data file.

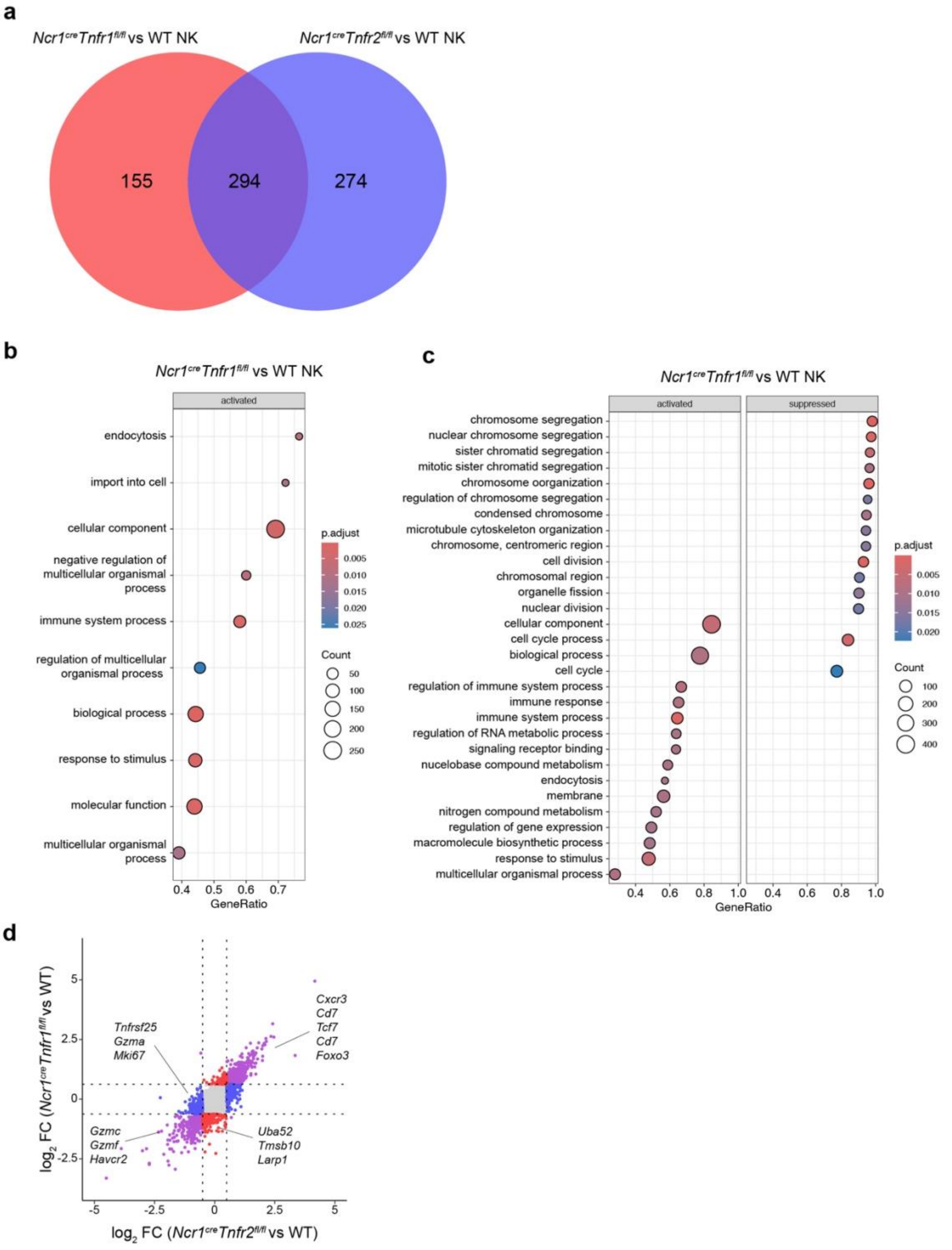

Supplementary Fig. 12: Comparison of differentially expressed genes between *$Ncr1^{cre}Tnfr1^{fl/fl}$* and WT or *$Ncr1^{cre}Tnfr2^{fl/fl}$* and WT. **a**, Venn diagram comparing number of differentially expressed genes between WT and *$Ncr1^{cre}Tnfr1^{fl/fl}$* or WT and *$Ncr1^{cre}Tnfr2^{fl/fl}$*, or shared between each analysis. **b,c**, GSEA analysis of DEGs between WT and *$Ncr1^{cre}Tnfr1^{fl/fl}$* (**b**) or WT and *$Ncr1^{cre}Tnfr2^{fl/fl}$* (**c**) by gseG. **d**, Comparison of log_2FC differentially expressed genes between WT and *$Ncr1^{cre}Tnfr1^{fl/fl}$*, and WT and *$Ncr1^{cre}Tnfr2^{fl/fl}$*. Dotted lines represent log_2FC cut-off of 0.5. Grey dots represent genes not differentially expressed, purple dots represent genes differentially expressed in both genotypes, red dots represent genes differentially expressed only in *$Ncr1^{cre}Tnfr1^{fl/fl}$* NK cells, and blue dots represent genes differentially expressed only in *$Ncr1^{cre}Tnfr2^{fl/fl}$* NK cells.

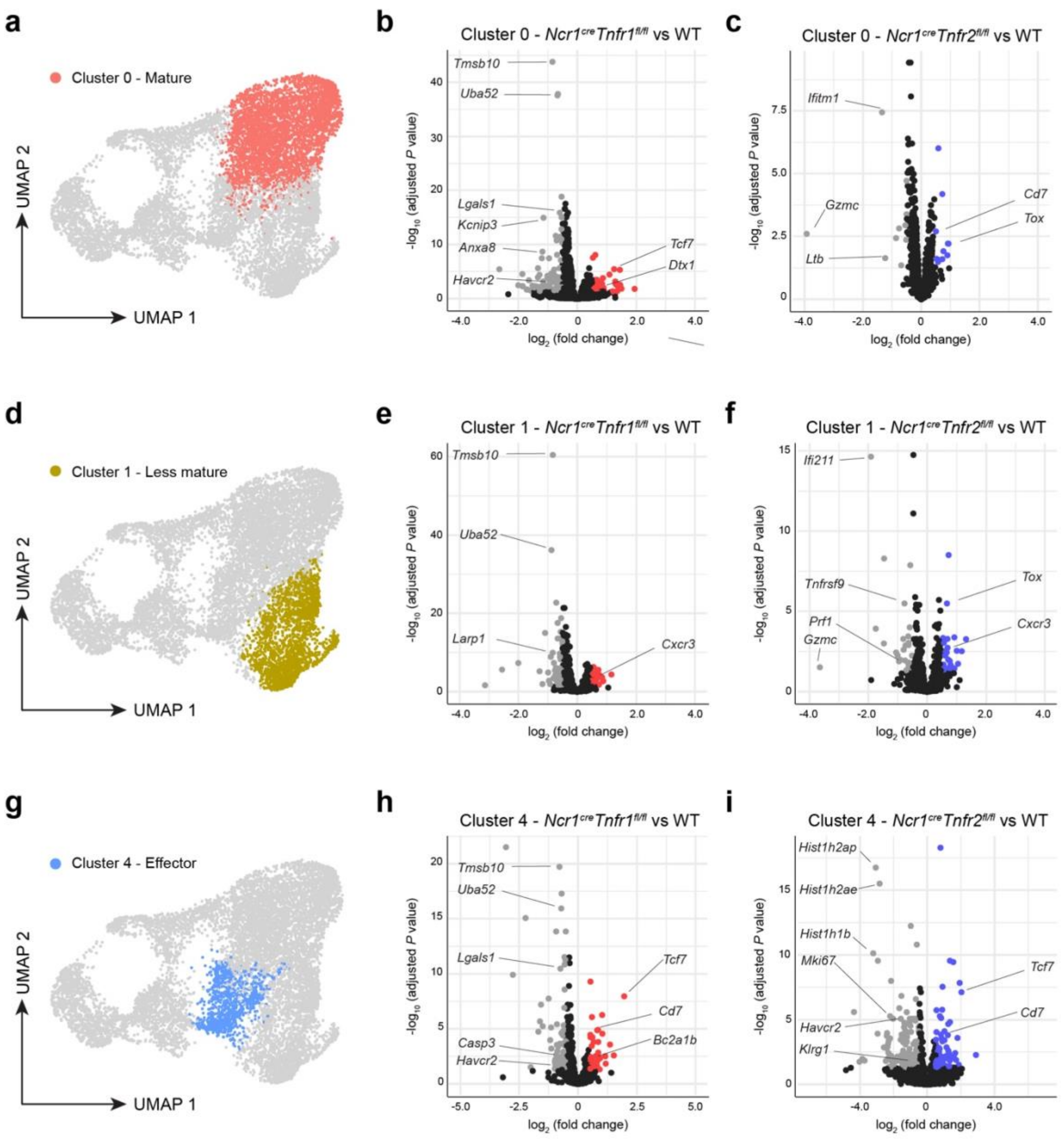

Supplementary Fig. 13: Differential gene expression between *Ncr1*^{cre}*Tnfr1*$^{fl/fl}$ and WT or *Ncr1*^{cre}*Tnfr2*$^{fl/fl}$ and WT across non-proliferating clusters. **a**, Representative UMAP highlighting the mature cluster. **b,c,** Volcano plots showing differentially expressed genes between WT and *Ncr1*^{cre}*Tnfr1*$^{fl/fl}$ NK cells, or WT and *Ncr1*^{cre}*Tnfr2*$^{fl/fl}$ NK cells from the mature NK cell cluster. **d**, Representative UMAP highlighting the less mature cluster. **e,f,** Volcano plots showing differentially expressed genes between WT and *Ncr1*^{cre}*Tnfr1*$^{fl/fl}$ NK cells, or WT and *Ncr1*^{cre}*Tnfr2*$^{fl/fl}$ NK cells from the less mature NK cell cluster. **g**, Representative UMAP highlighting the effector cluster. **h,i,** Volcano plots showing differentially expressed genes between WT and *Ncr1*^{cre}*Tnfr1*$^{fl/fl}$ NK cells, or WT and *Ncr1*^{cre}*Tnfr2*$^{fl/fl}$ NK cells from the effector NK cell cluster. For volcano plots, grey dots represent genes upregulated in WT cells, red dots represent genes upregulated in *Ncr1*^{cre}*Tnfr1*$^{fl/fl}$ cells, and blue dots represent genes upregulated in *Ncr1*^{cre}*Tnfr2*$^{fl/fl}$ cells ($log_2FC > 0.5$ and adjusted $P < 0.05$). Data from a single experiment (n = 4 *Ncr1*^{cre}, 4 *Ncr1*^{cre}*Tnfr1*$^{fl/fl}$, and 3 *Ncr1*^{cre}*Tnfr2*$^{fl/fl}$). Groups were compared using Wald test with Benjamini and Hochberg adjustment, where $P < 0.05$ was considered statistically significant.

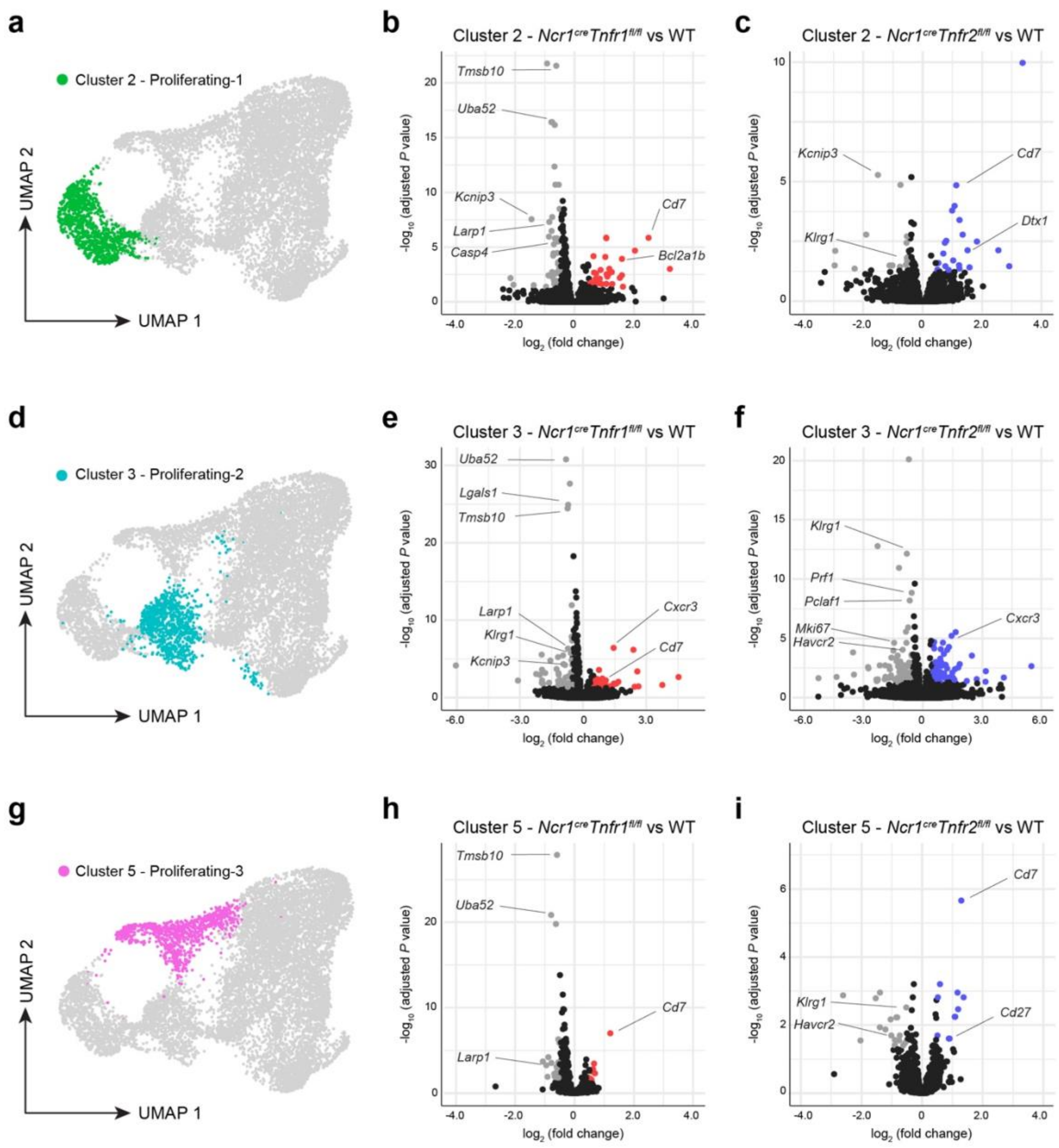

Supplementary Fig. 14: Differential gene expression between $Ncr1^{cre}Tnfr1^{fl/fl}$ and WT or $Ncr1^{cre}Tnfr2^{fl/fl}$ and WT across proliferating clusters. **a**, Representative UMAP highlighting the proliferating-1 cluster. **b,c,** Volcano plots showing differentially expressed genes between WT and $Ncr1^{cre}Tnfr1^{fl/fl}$ NK cells, or WT and $Ncr1^{cre}Tnfr2^{fl/fl}$ NK cells from the proliferating-1 NK cell cluster. **d**, Representative UMAP highlighting the proliferating-1 cluster. **e,f,** Volcano plots showing differentially expressed genes between WT and $Ncr1^{cre}Tnfr1^{fl/fl}$ NK cells, or WT and $Ncr1^{cre}Tnfr2^{fl/fl}$ NK cells from the proliferating-1 NK cell cluster. **g**, Representative UMAP highlighting the proliferating-1 cluster. **h,i,** Volcano plots showing differentially expressed genes between WT and $Ncr1^{cre}Tnfr1^{fl/fl}$ NK cells, or WT and $Ncr1^{cre}Tnfr2^{fl/fl}$ NK cells from the proliferating-1 NK cell cluster. For volcano plots, grey dots represent genes upregulated in WT cells, red dots represent genes upregulated in $Ncr1^{cre}Tnfr1^{fl/fl}$ cells, and blue dots represent genes upregulated in $Ncr1^{cre}Tnfr2^{fl/fl}$ cells ($log_2FC > 0.5$ and adjusted $P < 0.05$). Data from a single experiment (n = 4 $Ncr1^{cre}$, 4 $Ncr1^{cre}Tnfr1^{fl/fl}$, and 3 $Ncr1^{cre}Tnfr2^{fl/fl}$). Groups were compared using Wald test with Benjamini and Hochberg adjustment, where $P < 0.05$ was considered statistically significant.

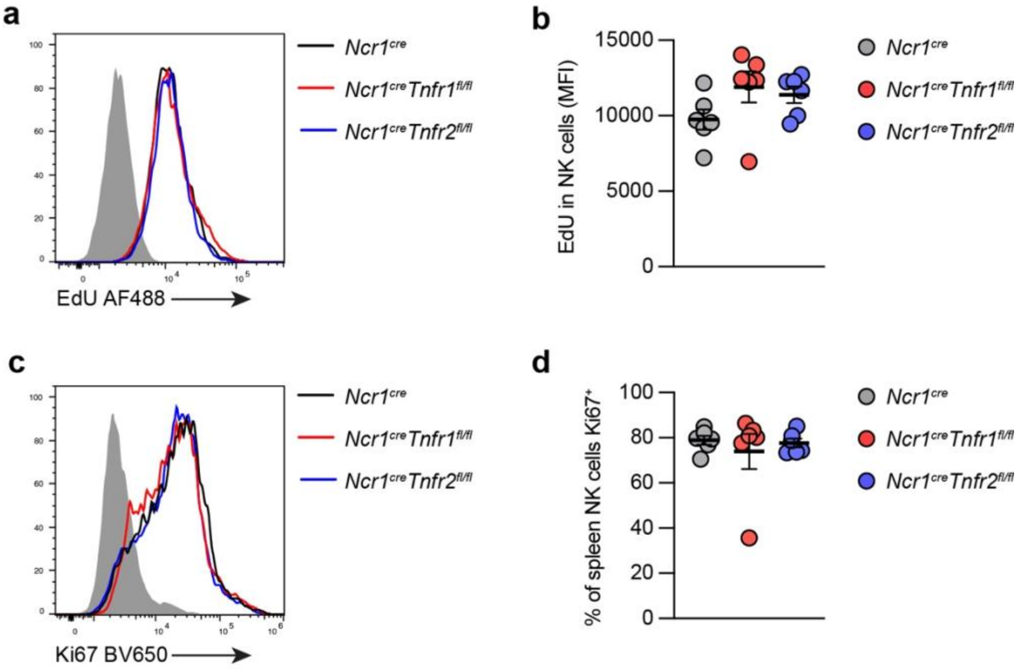

Supplementary Fig. 15: EdU incorporation and Ki67 expression in TNFR1 and TNFR2 deficient NK cells. Transgenic *Ncr1^{cre}*, *Ncr1^{cre}Tnfr1$^{fl/fl}$*, and *Ncr1^{cre}Tnfr2$^{fl/fl}$* mice were infected with *S*. Typhimurium and spleens and analyzed at day four post infection by flow cytometry. **a**, Representative histograms of EdU incorporation in NK cells. **b**, Relative levels of EdU in NK cells. **c,** Representative histograms of Ki67 expression in NK cells. **d**, Relative levels of Ki67 expression in NK cells. Data from **a-b** are from a single experiment (*n* = 6 biological replicates), data from **c-d** from a single experiment representative of two independent experiments (*n* = 6 biological replicates). Each dot represents one animal, error bars indicate mean ± SEM. Groups were compared using and Kruskal-Wallis test with Dunn's multiple comparisons test, where $P < 0.05$ was considered statistically significant. Source data are provided as a Source Data file.

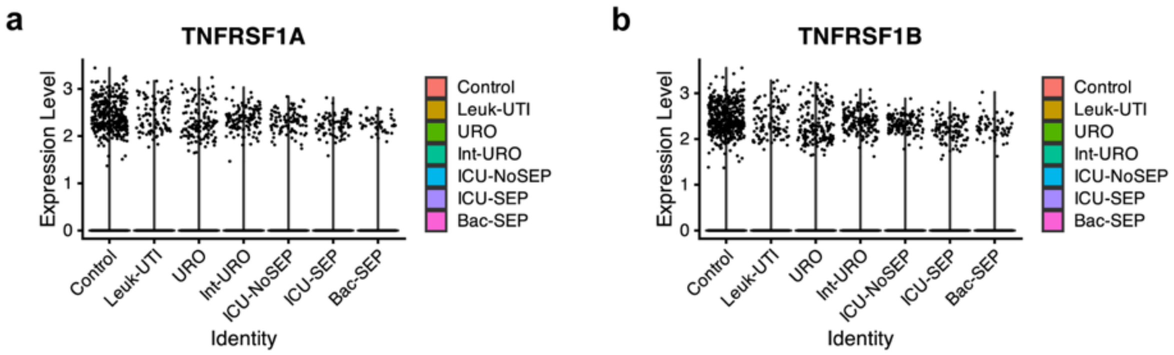

Supplementary Fig. 16: TNFR expression is similar in NK cells across sepsis patients. Reanalysis of scRNA-seq datasets of sepsis patients from Reyes et al, 2020. **a**, Expression of *TNFRSF1A* (encoding TNFR1) in NK cells across the cohorts. **b**, Expression of *TNFRSF1B* (encoding TNFR2) in NK cells across the cohorts. All data from published datasets (*n* = 19 control, 10 Leuk-UTI, 7 Int-URO, 9 URO, 7 ICU-NoSEP, 8 ICU-SEP, and 4 Bac-SEP). Abbreviations: UTI, urinary tract infection; URO, urosepsis; ICU, intensive care unit; SEP, sepsis; Bac, bacterial.

Supplementary Table 1: List of antibodies used in the study

Antibody	Clone	Supplier	Cat#	Dilution
Anti-CD45 BUV395	30-F11	BD Biosciences	565967	1:400
Anti-CD3e BUV737	145-2C11	BD Biosciences	612803	1:200
Anti-CD4 BUV496	GK1.5	BD Biosciences	6564667	1:400
Anti-CD8a BUV805	53-6.7	BD Biosciences	564920	1:400
Anti-NK1.1 BV510	PK136	BD Biosciences	563096	1:200
Anti-NK1.1 APC-Cy7	PK136	BD Biosciences	560618	1:200
Anti-TIGIT BV421	1G9	BD Biosciences	565270	1:200
Anti-CD11b BUV661	M1/70	BD Biosciences	565080	1:100
Anti-CD11b BV711	M1/70	BD Biosciences	563168	1:100
Anti-CD69 BV480	H1.2F3	BD Biosciences	746813	1:100
Anti-NKG2A/C/E BV605	20d5	BD Biosciences	564382	1:200
Anti-CD226 PE	TX42.1	BD Biosciences	567357	1:100
Anti-CD49b BV786	HMα2	BD Biosciences	740895	1:400
Anti-Tim3 APC	5D12	BD Biosciences	567164	1:200
Anti-Tim3 BB515	5D12	BD Biosciences	567810	1:400
Anti-KLRG1 BV421	2F1	BD Biosciences	562897	1:100
Anti-KLRG1 BV605	2F1	BD Biosciences	564013	1:200
Anti-Tim3 FITC	RMT3-23	eBioscience	11-5870-82	1:400
Anti-NKp46 Alexa Fluor 647	29A1.4	Biolegend	137628	1:100
Anti-NKp46 PE-Cy7	29A1.4	eBioscience	25-3351-82	1:100
Anti-CD107a PE	1D4B	Biolegend	121612	1:50
Anti-CD49a PE	HMα1	Biolegend	142604	1:100
Anti-CD49a BV711	HMα1	BD Biosciences	564863	1:100
Anti-CD120a/TNFR1 APC	55R-286	Biolegend	113006	1:100
Anti-CD120b/TNFR2 PE	TR75-89	Biolegend	113406	1:100
Anti-CD3e Biotin	145-2C11	Biolegend	100304	1:100
Anti-CD19 Biotin	MB19-1	Biolegend	101504	1:100
Anti-F4/80 Biotin	BM8	Biolegend	123106	1:100
Anti-Ly6G Biotin	1A8	Biolegend	127604	1:100
Anti-IFN-γ APC	XMG1.2	BD Biosciences	554413	1:50
Anti-Ki-67 BV650	B56	BD Biosciences	567122	1:200
Anti-FoxP3 PE-Cy5	JFK-16S	eBioscience	15-5773-82	1:400
Anti-Eomes PE-cf610	Dan11mag	eBioscience	61-4875-82	1:200
