# Supplementary material for: Dichotomous outcomes of TNFR1 and TNFR2 signaling in NK cell-mediated immune responses during inflammation
Source: Nat Commun. 2024 Nov 14;15:9871. doi: 10.1038/s41467-024-54232-y (PMC11564688; doi:10.1038/s41467-024-54232-y)
Supplement: Supplementary file 2 — Description of Additional Supplementary Files [file 41467_2024_54232_MOESM2_ESM.pdf]

# Description of Additional Supplementary Files

## Dichotomous outcomes of TNFR1 and TNFR2 signaling in NK cell-mediated immune responses during inflammation

Timothy R. McCulloch[#*], Gustavo R. Rossi[#], Louisa Alim[#], Pui Yeng Lam, Joshua Wong, Elaina Coleborn, Snehlata Kumari, Colm Keane, Andrew J. Kueh, Marco J. Herold, Christoph Wilhelm, Percy A. Knolle, Lawrence Kane, Timothy J. Wells, and Fernando Souza-Fonseca-Guimaraes[*]

[#] These authors contributed equally

[*] Correspondence: Fernando Souza-Fonseca-Guimaraes and Timothy R. McCulloch

E-mails: f.guimaraes@uq.edu.au and timothym@uni-bonn.de

Address: 37 Kent St, Woolloongabba, QLD 4102, Australia

**Supplementary Data 1: Differentially expressed genes between *Ncr1*$^{cre}$*Tnfr1*$^{fl/fl}$ and WT NK cells.** Transgenic *Ncr1*$^{cre}$ (WT) and *Ncr1*$^{cre}$*Tnfr1*$^{fl/fl}$ mice were infected with *S*. Typhimurium and NK cells analyzed by scRNA-seq on day four post-infection. Pseudobulk replicates were used to calculate differential gene expression using DESeq2. Data are from a single experiment (*n* = 4 *Ncr1*$^{cre}$ and 4 *Ncr1*$^{cre}$*Tnfr1*$^{fl/fl}$). Groups were compared using Wald test with Benjamini and Hochberg adjustment

**Supplementary Data 2: Differentially expressed genes between *Ncr1*$^{cre}$*Tnfr2*$^{fl/fl}$ and WT NK cells.** Transgenic *Ncr1*$^{cre}$ (WT) and *Ncr1*$^{cre}$*Tnfr2*$^{fl/fl}$ mice were infected with *S*. Typhimurium and NK cells analyzed by scRNA-seq on day four post-infection. Pseudobulk replicates were used to calculate differential gene expression using DESeq2. Data are from a single experiment (*n* = 4 *Ncr1*$^{cre}$ and 3 *Ncr1*$^{cre}$*Tnfr2*$^{fl/fl}$). Groups were compared using Wald test with Benjamini and Hochberg adjustment
