# Supplementary material for: Dichotomous outcomes of TNFR1 and TNFR2 signaling in NK cell-mediated immune responses during inflammation
Source: Nat Commun. 2024 Nov 14;15:9871. doi: 10.1038/s41467-024-54232-y (PMC11564688; doi:10.1038/s41467-024-54232-y)
Supplement: Supplementary file 5 — Reporting Summary [file 41467_2024_54232_MOESM5_ESM.pdf]

nature portfolio

Corresponding author(s): Timothy McCulloch, Fernando Guimaraes

Last updated by author(s): 2024/09/31

Reporting Summary

Nature Portfolio wishes to improve the reproducibility of the work that we publish. This form provides structure for consistency and transparency in reporting. For further information on Nature Portfolio policies, see our Editorial Policies and the Editorial Policy Checklist.

Statistics

For all statistical analyses, confirm that the following items are present in the figure legend, table legend, main text, or Methods section.

n/a	Confirmed	
☐	☒	The exact sample size (*n*) for each experimental group/condition, given as a discrete number and unit of measurement
☐	☒	A statement on whether measurements were taken from distinct samples or whether the same sample was measured repeatedly
☐	☒	The statistical test(s) used AND whether they are one- or two-sided<br>*Only common tests should be described solely by name; describe more complex techniques in the Methods section.*
☒	☐	A description of all covariates tested
☐	☒	A description of any assumptions or corrections, such as tests of normality and adjustment for multiple comparisons
☐	☒	A full description of the statistical parameters including central tendency (e.g. means) or other basic estimates (e.g. regression coefficient) AND variation (e.g. standard deviation) or associated estimates of uncertainty (e.g. confidence intervals)
☐	☒	For null hypothesis testing, the test statistic (e.g. *F*, *t*, *r*) with confidence intervals, effect sizes, degrees of freedom and *P* value noted<br>*Give P values as exact values whenever suitable.*
☒	☐	For Bayesian analysis, information on the choice of priors and Markov chain Monte Carlo settings
☒	☐	For hierarchical and complex designs, identification of the appropriate level for tests and full reporting of outcomes
☒	☐	Estimates of effect sizes (e.g. Cohen's *d*, Pearson's *r*), indicating how they were calculated

Our web collection on statistics for biologists contains articles on many of the points above.

Software and code

Policy information about availability of computer code

Data collection	FACS Diva v8.0.1 for flow cytometry data acquisition; SpectroFlo v2.2 for flow cytometry data acquisition.
Data analysis	Flow cytometry data were analysed in FlowJo v10.9; data visualization and statistical analysis was performed in GraphPad Prism v9.<br><br>scRNA-seq data was initially processed using CellRanger v7.1.0 and aligned to the GRCm38 (mm10) mouse reference genome. Subsequent analysis was performed in RStudio (2021.09.0-351) using R v4.2.2. The following R packages were used: ggplot v3.4.0; ggrepel v0.9.2; Seurat v4.3.0; SeuratObject v4.1.3; SingleCellExperiment v1.20.0; pheatmap v 1.0.12; miloR v1.2.0; escape v1.8.0; DESeq2 v1.40.2.

For manuscripts utilizing custom algorithms or software that are central to the research but not yet described in published literature, software must be made available to editors and reviewers. We strongly encourage code deposition in a community repository (e.g. GitHub). See the Nature Portfolio guidelines for submitting code & software for further information.

Data

Policy information about availability of data

All manuscripts must include a data availability statement. This statement should provide the following information, where applicable:

- Accession codes, unique identifiers, or web links for publicly available datasets
- A description of any restrictions on data availability
- For clinical datasets or third party data, please ensure that the statement adheres to our policy

The scRNA-seq datasets generated from this study have been deposited in the GEO repository database under the accession number GSE233790 (https://www.ncbi.nlm.nih.gov/geo/query/acc.cgi?acc=GSE233790). Published datasets from Ben-Moshe et al.31 can be accessed from the GEO repository database under the accession number GSE122084 (https://www.ncbi.nlm.nih.gov/geo/query/acc.cgi?acc=GSE122084), and dataset from Reyes et al.32 can be accessed from the Broad Institute Single Cell Portal under number SCP548 (https://singlecell.broadinstitute.org/single_cell/study/SCP548/an-immune-cell-signature-of-bacterial-sepsis-patient-pbmcs).

Research involving human participants, their data, or biological material

Policy information about studies with human participants or human data. See also policy information about sex, gender (identity/presentation), and sexual orientation and race, ethnicity and racism.

Reporting on sex and gender	N/A
Reporting on race, ethnicity, or other socially relevant groupings	N/A
Population characteristics	N/A
Recruitment	N/A
Ethics oversight	N/A

Note that full information on the approval of the study protocol must also be provided in the manuscript.

Field-specific reporting

Please select the one below that is the best fit for your research. If you are not sure, read the appropriate sections before making your selection.

☒ Life sciences ☐ Behavioural & social sciences ☐ Ecological, evolutionary & environmental sciences

For a reference copy of the document with all sections, see nature.com/documents/nr-reporting-summary-flat.pdf

Life sciences study design

All studies must disclose on these points even when the disclosure is negative.

Sample size	No statistical methods were used to determine sample sizes. For scRNA-seq experiments, sample sizes were determined in consultation with bioinformatics experts. For all other experiments, sample sizes were determined by availability of transgenic animals and repeated to ensure validity of results.
Data exclusions	Exclusion criteria were pre-established before the start of the study based on observations in our previous work published in McCulloch et al. (Immunology 167 (1), 54-63). In this study we found some mice do not take on infection. These mice do no lose weight after challenge, and consistently have no detectable bacterial burdens or alterations in immune parameters. Thus, mice that had not lost weight after 24 hours post infection were considered to not have been infected, and were excluded from the study at this point.
Replication	Experiments were performed twice to confirm replication, except for the scRNA-seq experiments.
Randomization	As most of this work is using transgenic animals, no randomization was possible.
Blinding	No formal blinding was performed in this study as transgenic animals and models with visual phenotypic differences were used

Reporting for specific materials, systems and methods

We require information from authors about some types of materials, experimental systems and methods used in many studies. Here, indicate whether each material, system or method listed is relevant to your study. If you are not sure if a list item applies to your research, read the appropriate section before selecting a response.

Materials & experimental systems

n/a	Involved in the study	
☐	☒	Antibodies
☒	☐	Eukaryotic cell lines
☒	☐	Palaeontology and archaeology
☐	☒	Animals and other organisms
☒	☐	Clinical data
☒	☐	Dual use research of concern
☒	☐	Plants

Methods

n/a	Involved in the study	
☒	☐	ChIP-seq
☐	☒	Flow cytometry
☒	☐	MRI-based neuroimaging

Antibodies

Antibodies used

The following antibodies were used for flow cytometry:
Anti-CD45 BUV395 (clone 30-F11), BD Biosciences, Cat#565967, RRID: AB_2651134
Anti-CD3e BUV737 (clone 145-2C11), BD Biosciences, Cat#612803, RRID: AB_2738781
Anti-CD4 BUV496 (clone GK1.5), BD Biosciences, Cat#6564667, RRID: AB_2722549
Anti-CD8a BUV805 (clone 53-6.7), BD Biosciences, Cat#564920, RRID: AB_2716856
Anti-NK1.1 BV510 (clone PK136), BD Biosciences, Cat#563096, RRID: AB_2738002
Anti-NK1.1 APC-Cy7 (clone PK136), BD Biosciences, Cat#560618, RRID: AB_1727569
Anti-TIGIT BV421 (clone 1G9), BD Biosciences, Cat#565270, RRID: AB_2688007
Anti-CD11b BUV661 (clone M1/70), BD Biosciences, Cat#565080, RRID: AB_2722548
Anti-CD11b BV711 (clone M1/70), BD Biosciences, Cat#563168, RRID: AB_2716860
Anti-CD69 BV480 (clone H1.2F3), BD Biosciences, Cat#746813, RRID: AB_2744067
Anti-NKG2A/C/E BV605 (clone 20d5), BD Biosciences, Cat#564382, RRID: AB_2738782
Anti-CD226 PE (clone TX42.1), BD Biosciences, Cat#567357, Mouse reactivity validated by QC testing
Anti-CD49b BV786 (clone HMα2), BD Biosciences, Cat#740895, RRID: AB_2740543
Anti-Tim3 APC (clone 5D12), BD Biosciences, Cat#567164, Mouse reactivity validated by QC testing
Anti-KLRG1 BV605 (clone 2F1), BD Biosciences, Cat#564013, RRID: AB_2722497
Anti-Tim3 FITC (clone RMT3-23), eBioscience, Cat#11-5870-82, RRID: AB_2688129
Anti-NKp46 PE-Cy7 (clone 29A1.4), eBioscience, Cat#25-3351-82, RRID: AB_2573442
Anti-CD107a PE (clone 1D4B), Biolegend, Cat#121612, RRID: AB_2134487
Anti-CD49a PE (clone HMα1), Biolegend, Cat#142604, RRID: AB_10945158
Anti-CD49a BV711 (clone HMα1), BD Biosciences, Cat#564863, RRID:AB_2738987
Anti-CD120a/TNFR1 APC (clone 55R-286), Biolegend, Cat#113006, RRID: AB_2208779
Anti-CD120b/TNFR2 PE (clone TR75-89) , Biolegend, Cat#113406, RRID: AB_2206941
Anti-CD19 Biotin (clone MB19-1), Biolegend, Cat#101504, RRID: AB_312823
Anti-F4/80 Biotin (clone BM8), Biolegend, Cat#123106, RRID: AB_893501
Anti-Ly6G Biotin (clone 1A8), Biolegend, Cat#127604, RRID: AB_1186108
Anti-IFN-γ APC (clone XMG1.2), BD Biosciences, Cat#554413, RRID: AB_398551
Anti-Ki-67 BV650 (clone B56), BD Biosciences, Cat#567122, Mouse reactivity tested in development
Anti-FoxP3 PE-Cy5 (clone JFK-16S), eBioscience, Cat#15-5773-82, RRID: AB_468806
Anti-Eomes PE-cf610 (clone Dan11mag), eBioscience, Cat#61-4875-82, RRID: AB_2574614
Anti-Tim3 BB515 (clone 5D12), BD Biosciences, Cat#567164, Mouse reactivity validated by QC testing

Validation

The following antibodies were used for flow cytometry:

Anti-CD45 BUV395 (clone 30-F11), BD Biosciences, Cat#565967, RRID: AB_2651134
Anti-CD3e BUV737 (clone 145-2C11), BD Biosciences, Cat#612803, RRID: AB_2738781
Anti-CD4 BUV496 (clone GK1.5), BD Biosciences, Cat#6564667, RRID: AB_2722549
Anti-CD8a BUV805 (clone 53-6.7), BD Biosciences, Cat#564920, RRID: AB_2716856
Anti-NK1.1 BV510 (clone PK136), BD Biosciences, Cat#563096, RRID: AB_2738002
Anti-NK1.1 APC-Cy7 (clone PK136), BD Biosciences, Cat#560618, RRID: AB_1727569
Anti-TIGIT BV421 (clone 1G9), BD Biosciences, Cat#565270, RRID: AB_2688007
Anti-CD11b BUV661 (clone M1/70), BD Biosciences, Cat#565080, RRID: AB_2722548
Anti-CD11b BV711 (clone M1/70), BD Biosciences, Cat#563168, RRID: AB_2716860
Anti-CD69 BV480 (clone H1.2F3), BD Biosciences, Cat#746813, RRID: AB_2744067
Anti-NKG2A/C/E BV605 (clone 20d5), BD Biosciences, Cat#564382, RRID: AB_2738782
Anti-CD226 PE (clone TX42.1), BD Biosciences, Cat#567357, Mouse reactivity validated by QC testing
Anti-CD49b BV786 (clone HMα2), BD Biosciences, Cat#740895, RRID: AB_2740543
Anti-Tim3 APC (clone 5D12), BD Biosciences, Cat#567164, Mouse reactivity validated by QC testing
Anti-KLRG1 BV605 (clone 2F1), BD Biosciences, Cat#564013, RRID: AB_2722497
Anti-Tim3 FITC (clone RMT3-23), eBioscience, Cat#11-5870-82, RRID: AB_2688129
Anti-NKp46 PE-Cy7 (clone 29A1.4), eBioscience, Cat#25-3351-82, RRID: AB_2573442
Anti-CD107a PE (clone 1D4B), Biolegend, Cat#121612, RRID: AB_2134487
Anti-CD49a PE (clone HMα1), Biolegend, Cat#142604, RRID: AB_10945158
Anti-CD49a BV711 (clone HMα1), BD Biosciences, Cat#564863, RRID:AB_2738987
Anti-CD120a/TNFR1 APC (clone 55R-286), Biolegend, Cat#113006, RRID: AB_2208779
Anti-CD120b/TNFR2 PE (clone TR75-89) , Biolegend, Cat#113406, RRID: AB_2206941
Anti-CD19 Biotin (clone MB19-1), Biolegend, Cat#101504, RRID: AB_312823
Anti-F4/80 Biotin (clone BM8), Biolegend, Cat#123106, RRID: AB_893501

Anti-Ly6G Biotin (clone 1A8), Biolegend, Cat#127604, RRID: AB_1186108
Anti-IFN-γ APC (clone XMG1.2), BD Biosciences, Cat#554413, RRID: AB_398551
Anti-Ki-67 BV650 (clone B56), BD Biosciences, Cat#567122, Mouse reactivity tested in development Anti-FoxP3 PE-Cy5 (clone JFK-16S), eBioscience, Cat#15-5773-82, RRID: AB_468806
Anti-Eomes PE-cf610 (clone Dan11mag), eBioscience, Cat#61-4875-82, RRID: AB_2574614
Anti-Tim3 BB515 (clone 5D12), BD Biosciences, Cat#567164, Mouse reactivity validated by QC testing

Animals and other research organisms

Policy information about studies involving animals; ARRIVE guidelines recommended for reporting animal research, and Sex and Gender in Research

Laboratory animals	Wild-type C57BL/6 mice were purchased from the Animal Resources Centre (Western Australia). Ncr1cre mice were crossed with Tim3fl/fl mice or FSF-Tim3 mice to generate novel strains with condition deletion (Ncr1creTim3fl/fl) or overexpression (Ncr1creFSF-Tim3) of Tim3 specifically within NK cells. Ncr1cre mice were also crossed with Tnfr1fl/fl and/or Tnfr2fl/fl mice to generate novel strains with condition deletion of TNFR1 (Ncr1creTnfr1fl/fl), TNFR2 (Ncr1creTnfr2fl/fl), or both TNFRs (Ncr1creTnfr1fl/flTnfr2fl/fl) specifically within NK cells. Ncr1cre/wt were used as wild-type controls for transgenic mouse experiment. Rag2-/-γc-/- were purchased from The Walter and Eliza Hall Institute for Medical Research. All transgenic mice were developed on the C57BL/6 background. Age (8-16 weeks) and sex matched mice were used for experimental purposes.
Wild animals	No wild animals were used.
Reporting on sex	Mice were sex matched in all experiments to prevent confounding factors.
Field-collected samples	No samples were collected from the field
Ethics oversight	All animal experiments were conducted according to approval by the University of Queensland Health Science Animal Ethics Committee (approval numbers UQDI/536/19, 2021/AE000584, and 2021/AE000585) and procedures were carried out in accordance with the regulatory standards of the National Health and Medical Research Council (NHMRC) and the Australian Code for the Responsible Conduct of Research.

Note that full information on the approval of the study protocol must also be provided in the manuscript.

Flow Cytometry

Plots

Confirm that:

☒ The axis labels state the marker and fluorochrome used (e.g. CD4-FITC).

☒ The axis scales are clearly visible. Include numbers along axes only for bottom left plot of group (a 'group' is an analysis of identical markers).

☒ All plots are contour plots with outliers or pseudocolor plots.

☒ A numerical value for number of cells or percentage (with statistics) is provided.

Methodology

Sample preparation	At experimental endpoints, mice were euthanized by CO2 asphyxiation and spleens and/or livers harvested for processing for flow cytometry. Spleens and livers were harvested and single cell suspensions generating by passing organ through 70 µm or 100 µm cell strainers, respectively. Liver immune cells were further enriched using 37.5% Percoll (GE Healthcare). Red blood cells (RBC) were lysed by incubation in RBC Lysis Buffer (Biolegend). Single cell suspensions were then washed and resuspended in FACS buffer for flow cytometry staining.
Instrument	Cytek Aurora 5L, BD FACSymphony A5, BD FACSAria Fusion
Software	FACS Diva v8.0.1 for flow cytometry data acquisition; SpectroFlo v2.2 for flow cytometry data acquisition; FlowJo v10.9 for data analysis.
Cell population abundance	Post-sort purities were determined by sampling post-sort fractions, which were consistently >95%. Example given in Figure S1b.
Gating strategy	Lymphocytes were gated by FSC-A vs SSC-A to exclude debris and unwanted populations. Doublets were excluded on a FSC-A vs FSC-H plot. Viable cells were then selected by excluding viability dye positive cells (PI or FVS440UV). CD45+ cells were taken to include all leukocytes. In some cases, lineage+ cells were excluded (CD19, F4/80, Ly6G). Various markers were then used to identify relevant immune populations. Positive and negative gating was determined by eye for markers with clear separation, and FMO controls used for others.

☒ Tick this box to confirm that a figure exemplifying the gating strategy is provided in the Supplementary Information.
